# Plasma aldosterone concentration and plasma renin activity in untreated hypertensive patients: gender differences and therapeutic implications

**DOI:** 10.1038/s41440-026-02666-w

**Published:** 2026-05-20

**Authors:** Masafumi Nakayama, Yoshihiro Miyamoto, Takeshi Morimoto, Yuichiro Arima, Kenichi Tsujita

**Affiliations:** 1 Nakayama Cardiovascular Clinic, Amakusa, Japan; 2 National Cerebral and Cardiovascular Center, Suita, Japan; 3 Department of Data Science, Hyogo Medical University, Nishinomiya, Japan; 4 Department of Anatomy, Faculty of Life Sciences, Kumamoto University, Kumamoto, Japan; 5 Department of Cardiovascular Medicine, Graduate School of Medical Sciences, Kumamoto University, Kumamoto, Japan

**Keywords:** Implemental hypertension, Renin, Aldosterone, Sodium, Gender differences

## Abstract

The renin–angiotensin–aldosterone system regulates blood pressure and is influenced by antihypertensive therapy. We examined plasma aldosterone concentration (PAC), plasma renin activity (PRA), and clinical characteristics in untreated hypertensive patients without primary aldosteronism, followed for 3 years. Among 456 newly diagnosed patients, 397 (219 males, 178 females) were analyzed after excluding those with low blood pressure, suspected primary aldosteronism, or renovascular hypertension. PAC and PRA were measured at baseline, and clinic blood pressure was monitored at 3 months, 1 year, and 3 years. A high PAC/PRA ratio (>200) was found in 19.6% of patients and was significantly associated with female sex (*p* < 0.0001) and higher serum sodium (*p* = 0.0004). PAC did not differ between groups, but PRA was markedly lower in the high PAC/PRA group (*p* < 0.0001). Multivariate analysis identified female sex as the only independent predictor (*p* < 0.0001), with prevalence rates of 29.8% in females and 11.4% in males. In females, PAC/PRA correlated positively with sodium, while PRA correlated negatively, relationships not observed in males. Mineralocorticoid receptor antagonists (MRAs) were used as first-line therapy in patients with high PAC/PRA. Their blood pressure declined from 159/95 mmHg at baseline to 137/80, 135/78, and 133/77 mmHg at 3 months, 1 year, and 3 years, respectively. In the normal group, values were 159/96, 142/81, 138/77, and 136/77 mmHg, with greater systolic reduction at 3 months in the high PAC/PRA group (*p* = 0.03). We identified gender differences in the renin–aldosterone–sodium system. MRAs effectively reduced blood pressure in patients with relatively low PRA levels.

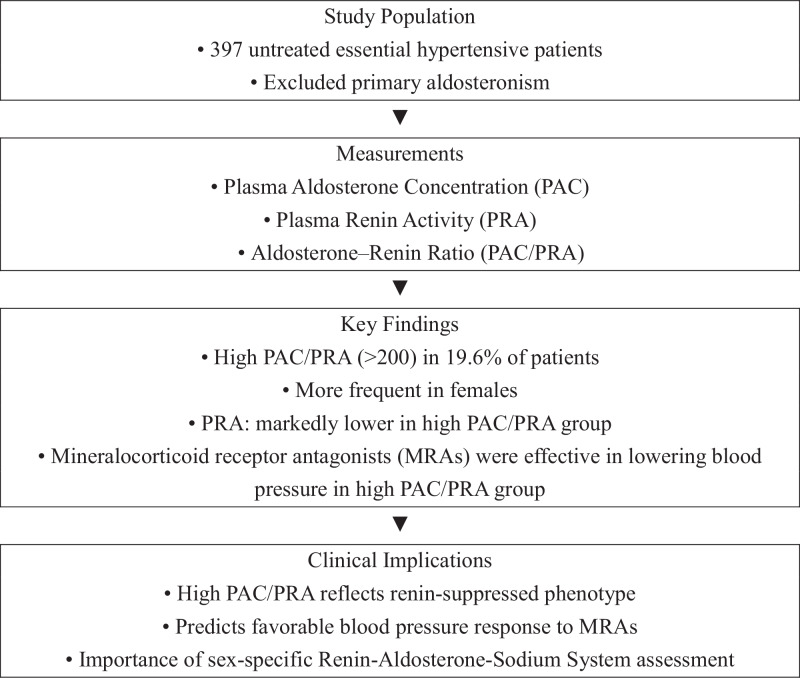

## Background

The renin–angiotensin–aldosterone system (RAAS) plays a crucial role in maintaining body fluid, sodium, and potassium homeostasis, and consequently, blood pressure. Traditionally, high sodium intake results in decreased plasma renin activity (PRA), plasma angiotensin II concentrations, and plasma aldosterone concentrations (PAC) [1–3].

Hypertension is an important risk factor for cardiovascular disease [4, 5]. Currently, several antihypertensive drugs target the renin–angiotensin–aldosterone system (RAAS), including renin inhibitors, angiotensin-converting enzyme inhibitors, angiotensin II receptor blockers, and mineralocorticoid receptor antagonists (MRAs). However, the choice of antihypertensive medication for patients with uncomplicated primary hypertension is largely left to the discretion of individual physicians. Although PRA and PAC can be useful indicators for selecting antihypertensive drugs in primary hypertension, most guidelines recommend measuring PRA and PAC primarily in patients with suspected secondary hypertension, particularly for screening for primary aldosteronism [6–9]. Classical studies have shown that a substantial proportion of patients with essential hypertension exhibit suppressed renin activity, a condition referred to as low-renin hypertension. These patients often demonstrate increased salt sensitivity and may respond favorably to natriuretic therapy or mineralocorticoid receptor antagonists. Previous investigators have therefore proposed that measuring renin activity could help guide the selection of antihypertensive therapy. However, most studies have focused on renin levels alone, and relatively few have examined the clinical significance of the PAC/PRA ratio in untreated hypertensive patients without primary aldosteronism [10–12]. Moreover, many antihypertensive drugs can influence RAAS. In this study, we analyzed the clinical characteristics, including PRA and PAC, in newly diagnosed hypertensive patients without primary aldosteronism who had not yet received antihypertensive treatment.

It remains unclear whether MRAs are recommended for patients with a high PAC/PRA ratio without primary aldosteronism. We administered MRAs as first-line therapy to patients with a high PAC/PRA ratio and followed their clinic blood pressure for three years.

We hypothesized that essential hypertension with a high PAC/PRA ratio exhibits distinct clinical characteristics that reflect differences in aldosterone–renin–sodium homeostasis. Furthermore, this study aimed to clarify the effects of mineralocorticoid receptor antagonists (MRAs) in patients with a high PAC/PRA ratio.

## Methods

### Study patients

This study enrolled patients who were treated at Nakayama Cardiovascular Clinic between April 2011 and March 2021. All patient data were anonymized at the clinic and subsequently sent to Hyogo Medical University for statistical analysis. The study results were independently evaluated at Kumamoto University and the National Cerebral and Cardiovascular Center. At their first clinic visit, patients received lifestyle guidance. Several weeks later, those who were strongly suspected of having hypertension based on both home and clinic blood pressure measurements were enrolled prior to the initiation of treatment. At enrollment, no participant was receiving β-blockers, calcium channel blockers, diuretics, steroids or other cardiovascular medications, including for arrhythmia or angina.

We measured plasma aldosterone concentration (PAC, pg/mL) and plasma renin activity (PRA, ng/mL/h) in 456 consecutive, newly diagnosed outpatients with hypertension who had never received antihypertensive medications. None of the patients had heart failure. Blood samples were collected after at least 30 min of bed rest. We excluded 28 cases with blood pressure <135/85 mmHg after this period of bed rest. We also excluded 28 hypertensive patients with high aldosterone concentrations (≥ 120 pg/mL) and a high PAC/PRA ratio (≥200), as all were suspected of having primary aldosteronism [6]. Additionally, 3 patients with distinct renovascular hypertension—diagnosed based on symptoms, medical history, electrocardiogram, abdominal ultrasonography, CT, and laboratory data—were excluded. In total, 397 patients were analyzed (219 males, mean age 56.5 ± 12.8 years; 178 females, mean age 61.3 ± 13.6 years). This study was approved by the Ethics Committee of Kumamoto University School of Medicine, and written informed consent was obtained from all patients.

### Follow-up study

We followed the study patients for 3 years. Clinic blood pressure was measured at 3 months, 1 year, and 3 years after enrollment. We measured blood pressure using an automated oscillometric device (OMRON Automatic Blood Pressure Monitor, Model HBP-9020, Omron Healthcare Co., Ltd., Kyoto, Japan) after 5 min of rest, repeated 2–3 times, and calculated the average of the last 2 measurements.

Patients were divided into high PAC/PRA ratio (>200) and normal groups. MRAs were administered as first-line therapy to the high PAC/PRA group. All patients were treated for hypertension according to the JSH 2019 guidelines [6], except for the use of MRAs.

### Measurement of PAC and PRA

Plasma aldosterone concentration was determined using a commercially available radioimmunoassay (RIA) (SPAC-S Aldosterone Kit; Fuji Rebio Co., Tokyo, Japan) according to the manufacturer’s instructions. Venous blood samples were collected in chilled EDTA-containing tubes after the patients had remained seated at rest for at least 15 min, immediately placed on ice, and centrifuged at 3000 rpm for 10 min at 4 °C. Calibration curves were generated using kit-supplied standards, and quality control samples were included in each run.

Plasma renin activity (PRA) was measured using either a radioimmunoassay or an enzyme immunoassay, as previously described [11]. Briefly, plasma samples were incubated at 37 °C to allow the enzymatic generation of angiotensin I, which was subsequently quantified using assay-specific antibodies. PRA was calculated and expressed as ng/mL/hour. All measurements were performed in duplicate, and internal quality control materials were included in each batch. When both assay methods were used during the study period, results were cross-validated using parallel samples to ensure analytical consistency.

### Statistical analyses

The primary comparison in this study was between patients with a PAC/PRA ratio ≥200 (high group) and those with a PAC/PRA ratio <200 (normal group). Patient characteristics are presented as mean ± standard deviation (SD), median (interquartile range [IQR]), or number and percentage. An unpaired *t-*test, Wilcoxon rank-sum test, or Chi-square test was used to compare patient characteristics based on the nature of variables. Considering that the distribution is skewed, the correlation between variables was assessed using Spearman’s rank correlation coefficient and estimated as ρ. The variables included in the correlations were PAC, PRA, PAC/PRA ratio, and sodium and potassium levels. We constructed multivariable logistic regression models to determine independent variables associated with the high group. Variables included in the logistic regression model were gender, age (≥58 vs. <58 years), body mass index (BMI) (≥24 vs. <24 kg/m^2^), current or past smoker, diabetes mellitus, and dyslipidemia. The thresholds for age and BMI were determined using median values. We used a full model without a variable selection procedure. Because sex was significantly associated with the high and normal groups, we conducted the same analyses separately for men and women, except for the multivariable logistic regression model. We also compared patients’ characteristics between male and female. All reported *p* values were two-tailed, and statistical significance was set at *p* < 0.05. Statistical analyses were performed using JMP 18.2 software (SAS Institute Inc., Cary, NC).

## Results

### Total population

**Fig. 1:**
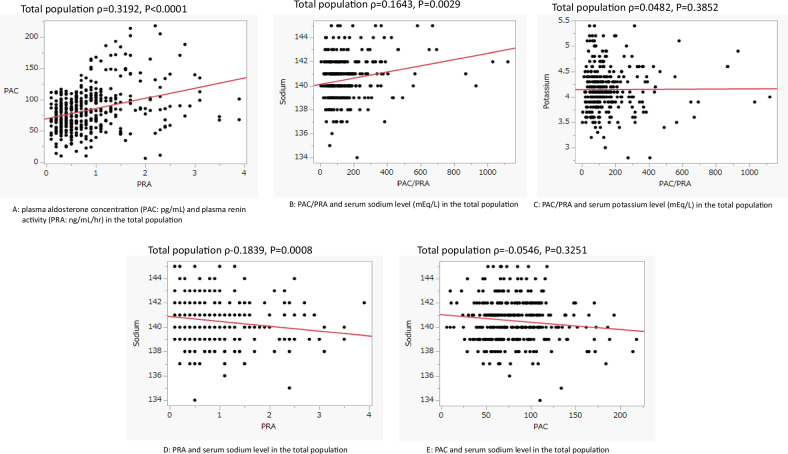
**A** Plasma aldosterone concentration (PAC, pg/mL) and plasma renin activity (PRA, ng/mL/hr) in the total population; **B** PAC/PRA ratio and serum sodium level (mEq/L) in the total population; **C** PAC/PRA ratio and serum potassium level (mEq/L) in the total population; **D** PRA and serum sodium level in the total population; **E** PAC and serum sodium level in the total population

There was a significant positive correlation between PRA and PAC (ρ = 0.3192, *p* < 0.0001) (Fig. 1A). The PAC/PRA ratio was significantly positively correlated with serum sodium levels (ρ = 0.1643, *p* < 0.0001) (Fig. 1B); however, no correlation was observed between the PAC/PRA ratio and serum potassium levels (Fig. 1C). Significant negative correlations were observed between PRA and serum sodium levels (ρ = −0.1839, *p* = 0.0008) (Fig. 1D). No significant correlation was observed between PAC and serum sodium levels (Fig. 1E). In this study, 10 patients had a serum potassium level below 3.5 mEq/L, of whom 4 were classified into the high PAC/PRA group.

### Predictive factors for high PAC/PRA ratio (>200)

In this study, 19.6% (78/397) of patients had a high PAC/PRA ratio (>200). We compared the clinical characteristics of the high PAC/PRA (>200) and normal groups (Table 1a). In univariate analyses, a high PAC/PRA ratio was significantly associated with female sex (*p* < 0.0001) and higher serum sodium levels (*p* = 0.0004); however, it was not significantly associated with other factors, including age, body mass index, smoking, diabetes mellitus, dyslipidemia, blood pressure, pulse rate, serum potassium levels, serum urea nitrogen, estimated glomerular filtration rate, or hemoglobin levels. There was no difference in PAC between the two groups, whereas PRA was significantly lower in the high PAC/PRA group than in the normal group (*p* < 0.0001). Consequently, the PAC/PRA ratio was significantly higher in the high PAC/PRA group (*p* < 0.0001). Multiple logistic regression analyses revealed that female sex was the only significant independent predictor of a high PAC/PRA ratio (*p* < 0.0001) (Table 1b). Subsequently, we analyzed differences between male and female patients.

**Table 1:** a. Univariate analysis. b. Multiple logistic regression analysis: independent factors associated with high PAC/PRA (≥200)

a.			
Characteristics	High group PAC/PRA ≥ 200	Normal group	*P* value
	(*n* = 78)	PAC/PRA < 200	
		(*n* = 319)	
Age - years, mean (SD)	60.6 (12.2)	58.2 (13.6)	0.15
Male, *n* (%)	25 (33)	194 (61)	<0.0001
Body Mass Index - kg/m^2^, median (IQR)	23.7(22.0–27.2)	23.9 (22.0–27.3)	0.96
Current smoker or past smoker, *n* (%)	10 (13)	60 (19)	0.21
Diabetes Mellitus, *n* (%)	5 (6)	22 (7)	0.88
Dyslipidemia, *n* (%)	8 (10)	42 (13)	0.49
Systolic Blood Pressure-mmHg, mean (SD)	159 (20)	159 (18)	0.89
Diastolic Blood Pressure-mmHg, mean (SD)	95 (12)	96 (13)	0.27
Pules rate - /minute, mean (SD)	68(14)	70 (13)	0.24
Sodium - mEq/L, mean (SD)	141 (2)	140 (2)	0.0004
Potassium - mEq/L, mean (SD)	4.1 (0.5)	4.1 (0.4)	0.92
Urea Nitrogen - mg/dL, mean (SD)	13.8(3.5)	14.4 (4.1)	0.27
Creatinine - mg/dL, mean (SD)	0.7 (0.1)	0.8 (0.2)	0.004
eGFR - mL/min/1.73 m^2^, mean (SD)	79.5 (17.9)	78.2 (17.8)	0.61
Red Blood Cells - /μL	434 (55)	443 (54)	0.21
Hemoglobin - mg/dL, mean (SD)	13.5 (1.5)	13.9 (1.7)	0.08
Hematocrit - %, mean (SD)	40.1 (4.8)	41.0 (5.0)	0.15
PAC - pg/mL, median (IQR)	84.7 (68.5–95.8)	81.3 (58.8–103.0)	0.54
PRA - ng/mL/hr, median (IQR)	0.30 (0.20–0.30)	0.90 (0.60–1.30)	<0.0001
PAC/PRA, median (IQR)	310.0 (247.5–386.6)	94.9 (63.0–133.8)	–

b.		
Factor	Odds ratio	*P* value
Male	0.277481 (0.146712–0.524808)	<0.0001
Age ≥ 59	1.473012 (0.786555–2.758566)	0.2263
BMI ≥ 24	1.424305 (0.763747–2.656176)	0.266
Current smoker or past smoker	0.925472 (0.347945–2.461595)	0.8767
Diabetes Mellitus	1.212621 (0.407781–3.605978)	0.7288
Dyslipidemia	0.695962 (0.295892–1.636959)	0.4062

### Comparison between the high PAC/PRA and normal groups in males and females

We compared the clinical characteristics of males in the high PAC/PRA (>200) and normal groups (Table 2a). No significant associations were observed between a high PAC/PRA ratio and clinical characteristics, including age, body mass index, smoking, diabetes mellitus, dyslipidemia, blood pressure, pulse rate, serum sodium level, serum potassium level, serum urea nitrogen level, serum creatinine level, and eGFR in males.

**Table 2:** a. Univariate analysis: males. b. Univariate analysis: females

a.			
Characteristics	High group PAC/PRA ≥ 200	Normal group	*P* value
	(*n* = 25)	PAC/PRA < 200	
		(*n* = 194)	
Age - years, mean (SD)	57.7 (11.0)	56.3 (13.0)	0.61
Body Mass Index - kg/m^2^, median (IQR)	25.4 (22.0–27.6)	25.1 (22.3–28.0)	0.69
Current smoker or past smoker, *n* (%)	6 (24)	49 (25)	0.89
Diabetes Mellitus, n (%)	3 (12)	15 (8)	0.44
Dyslipidemia, n (%)	2 (8)	19 (10)	1
Systolic Blood Pressure-mmHg, mean (SD)	157 (24)	150 (19)	0.55
Diastolic Blood Pressure-mmHg, mean (SD)	97 (11)	98 (13)	0.74
Pules rate - /minute, mean (SD)	66 (10)	70 (13)	0.15
Sodium - mEq/L, mean (SD)	141 (3)	140 (2)	0.41
Potassium - mEq/L, mean (SD)	4.2 (0.5)	4.2 (0.4)	0.61
Urea Nitrogen - mg/dL, mean (SD)	13.3 (3.2)	14.7 (4.1)	0.14
Creatinine - mg/dL, mean (SD)	0.8 (0.1)	0.8 (0.2)	0.34
eGFR - mL/min/1.73m^2^, mean (SD)	80.9 (15.5)	77.5 (16.7)	0.38
PAC - pg/mL, median (IQR)	84.7(70.3–101.5)	82.7 (59.3–103.0)	0.69
PRA - ng/mL/hr, median (IQR)	0.30 (0.25–0.40)	1.00 (0.70–1.50)	<0.0001
PAC/PRA, median (IQR)	255.0 (217.0–292.0)	82.1 (56.4–119.3)	–

b.			
Characteristics	High group PAC/PRA ≥ 200	Normal group	*P* value
	*(n* = 53)	PAC/PRA < 200	
		*(n* = 125)	
Age - years, mean (SD)	62.0 (12.6)	61.1 (14.0)	0.68
Body Mass Index - kg/m^2^, median (IQR)	23.5 (22.0–27.1)	22.7 (21.4–25.2)	0.11
Current smoker or past smoker, *n* (%)	4 (8)	11 (9)	1
Diabetes Mellitus, *n* (%)	2 (4)	7 (6)	1
Dyslipidemia, *n* (%)	6 (11)	23 (18)	0.24
Systolic Blood Pressure-mmHg, mean (SD)	160 (19)	159 (17)	0.75
Diastolic Blood Pressure-mmHg, mean (SD)	93 (13)	94 (12)	0.89
Pules Rate - /minute, mean (SD)	69 (15)	70 (13)	0.57
Sodium - mEq/L, mean (SD)	141 (2)	140 (2)	0.002
Potassium - mEq/L, mean (SD)	4.1 (0.5)	4.1 (0.4)	0.99
Urea Nitrogen - mg/dL, mean (SD)	14.1 (3.6)	14.0 (3.9)	0.95
Creatinine - mg/dL, mean (SD)	0.6 (0.2)	0.6 (0.2)	0.72
eGFR - mL/min/1.73 m^2^, mean (SD)	78.9 (18.9)	79.4 (19.2)	0.89
PAC - pg/mL, median (IQR)	84.9 (67.5–95.0)	77.3 (56.7–103.0)	0.45
PRA - ng/mL/hr, median (IQR)	0.20 (0.20–0.30)	0.70 (0.50–0.90)	<0.0001
PAC/PRA, median (IQR)	340.0 (281.5–429.0)	118.8 (74.7–156.0)	–

We also compared the clinical characteristics of females in the high PAC/PRA ratio ( >200) and normal groups (Table 2b). Serum sodium levels were significantly higher in the high PAC/PRA group than in the normal group (*p* = 0.002). No significant associations were observed between a high PAC/PRA ratio and other clinical characteristics, including age, body mass index, smoking, diabetes mellitus, dyslipidemia, blood pressure, pulse rate, serum potassium level, serum urea nitrogen level, serum creatinine level, and eGFR in females.

### Gender differences in clinical characteristics

We compared the clinical characteristics between males and females (Table 3). On univariate analysis, females were significantly older than males (*p* = 0.0003). The body mass index was significantly lower in females than in males (*p* = 0.0003). The incidence of smoking was significantly lower in females than in males (*p* < 0.0001). The incidence of dyslipidemia was significantly higher in females than in males (*p* = 0.045). The diastolic blood pressure was significantly lower in females than in males (*p* = 0.0006). Though serum creatinine levels were significantly lower in females than in males, there was no significant difference in the eGFR between females and males.

**Table 3:** Univariate analysis: gender differences

Characteristics	Male	Female	*P* value
	(*n* = 219)	(*n* = 178)	
Age - years, mean (SD)	56.5 (12.8)	61.3 (13.6)	0.0003
Body Mass Index - kg/m^2^, median (IQR)	25.1 (22.3–27.9)	22.9 (21.6–25.9)	0.0003
Current Smoker or Past Smoker, *n* (%)	55 (25)	15 (9)	<0.0001
Diabetes Mellitus, *n* (%)	18 (8)	9 (5)	0.21
Dyslipidemia, *n* (%)	21 (10)	29 (16)	0.045
Systolic Blood Pressure-mmHg, mean (SD)	159 (20)	159 (18)	0.97
Diastolic Blood Pressure-mmHg, mean (SD)	98 (13)	94 (12)	0.0006
Pules Rate - /minute, mean (SD)	69 (12)	70 (14)	0.73
Sodium - mEq/L, mean (SD)	140 (2)	141 (2)	0.07
Potassium - mEq/L, mean (SD)	4.2 (0.4)	4.1 (0.4)	0.09
Urea Nitrogen - mg/dL, mean (SD)	14.5 (4.0)	14.0 (3.8)	0.25
Creatinine - mg/dL, mean (SD)	0.8 (0.2)	0.6 (0.2)	<0.0001
eGFR - mL/min/1.73 m^2^, mean (SD)	77.9 (16.6)	79.2 (19.0)	0.49
PAC - pg/mL, median (IQR)	82.8 (60.5–103.0)	79.8 (61.9–102.0)	0.11
PRA - ng/mL/hr, median (IQR)	0.90 (0.50–1.40)	0.50 (0.30–0.80)	<0.0001
PAC/PRA, median (IQR)	92.5 (61.8–140.0)	146.0 (94.5–252.4)	–

There was no gender difference in PAC, whereas PRA was significantly lower in females than in males (*p* < 0.0001). As a result, the PAC/PRA ratio was significantly higher in females than in males (*p* < 0.0001). The prevalence of high PAC/PRA ratio (>200) was 29.8% (53/178) in females and 11.4% (25/219) in males.

### Correlation analyses for males and females

**Fig. 2:**
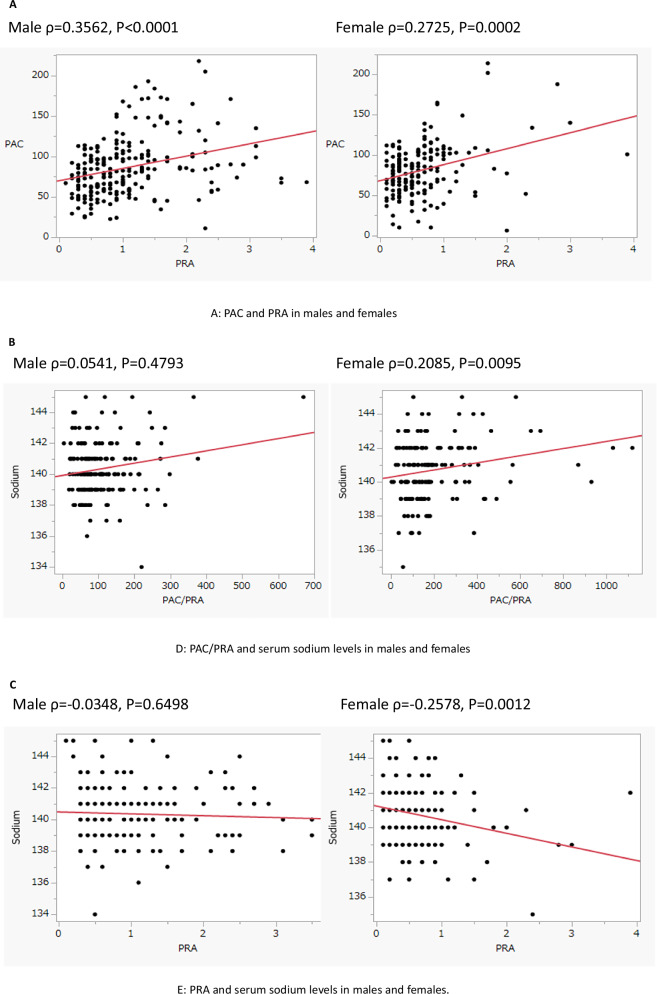
**A** Plasma aldosterone concentration (PAC) and plasma renin activity (PRA) in males and females; **B** PAC/PRA ratio and serum sodium levels in males and females; **C** PRA and serum sodium levels in males and females

There was a significant positive correlation between PRA and PAC in both genders (male: ρ = 0.3562, *p* < 0.0001; female: ρ = 0.2725, *p* = 0.0002) (Fig. 2A). In females, the PAC/PRA ratio was significantly positively correlated with serum sodium levels (ρ = 0.2085, *p* = 0.0095) (Fig. 2B), whereas no such correlation was observed in males. In females, PRA was significantly negatively correlated with serum sodium levels (ρ = −0.2578, *p* = 0.0012) (Fig. 2C), but no significant correlation was found in males.

### Follow-up study

We administered MRAs as first-line therapy to the high PAC/PRA group. All patients were treated for hypertension in accordance with the JSH 2019 guidelines [6], except for the use of MRAs. Table 4 shows antihypertensive medications at baseline (0 day), at 3 months, 1 year, and 3 years.

**Table 4:** Antihypertensive medications before therapy, at 3 months, 1 year, and 3 years

(a) Medication use — normal group and high PAC/PRA group						
Before therapy						
Group	*n*	ARBs/ACEI	Diuretics	CCBs	Beta-blocker	MRA
Normal group	319	0%	0%	0%	0%	0%
High PAC/PRA group	78	0%	0%	0%	0%	0%
At 3 months						
Normal group	265	72.50%	9.40%	33.20%	14.30%	29.10%
High PAC/PRA group	71	33.30%	0%	9.70%	7.00%	81.70%
At 1 Year						
Normal group	239	71.50%	10.00%	37.20%	18.80%	26.80%
High PAC/PRA group	69	53.60%	0%	18.80%	10.10%	81.20%
At 3 years						
Normal group	224	71.90%	11.60%	39.30%	17.90%	29.50%
High PAC/PRA group	56	46.40%	0%	21.40%	10.70%	80.40%
(b) Number of antihypertensive agents — normal group and high PAC/PRA group						
At 3 months						
Group	1-drug	2-drug	3-drug	4-drug		
Normal group	50.60%	41.50%	6.80%	1.10%		
High PAC/PRA group	49.30%	45.10%	5.60%	0%		
At 1 year						
Normal group	47.70%	41.40%	9.60%	1.30%		
High PAC/PRA group	46.40%	43.50%	10.10%	0%		
At 3 years						
Normal group	43.80%	42.90%	12.90%	0.40%		
High PAC/PRA group	50.00%	41.10%	8.90%	0%		

Values are expressed as percentages. Percentages were calculated using the number of patients in each group at each time point as the denominator

*PAC* plasma aldosterone concentration, *PRA* plasma renin activity, *ARB* angiotensin II receptor blocker, *ACEI* angiotensin-converting enzyme inhibitor, *CCB* calcium channel blocker, *MRA* mineralocorticoid receptor antagonist

**Fig. 3:**
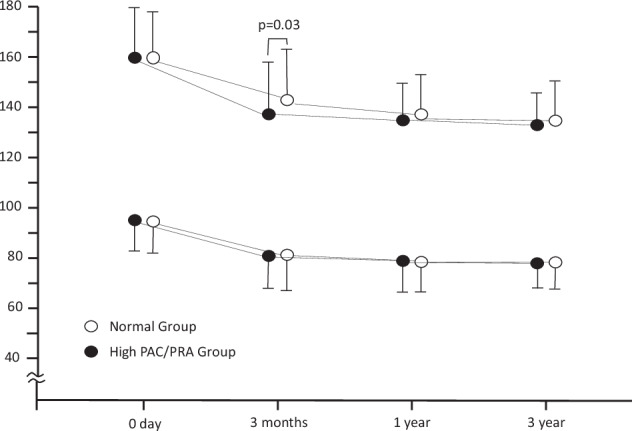
Changes in blood pressure at baseline (0 day), 3 months, 1 year, and 3 years in the high plasma aldosterone concentration (PAC)/plasma renin activity (PRA) group and the normal group

In the high PAC/PRA group, the mean blood pressure ( ± SD) at baseline, 3 months, 1 year, and 3 years was 159 ± 20/95 ± 12 mmHg, 137 ± 16/80 ± 12 mmHg, 135 ± 18/78 ± 13 mmHg, and 133 ± 18/77 ± 11 mmHg, respectively (Fig. 3). In the normal group, the corresponding values were 159 ± 18/96 ± 13 mmHg, 142 ± 19/81 ± 13 mmHg, 138 ± 18/77 ± 12 mmHg, and 136 ± 17/77 ± 12 mmHg, respectively. At 3 months after treatment, systolic blood pressure was significantly lower in the high PAC/PRA group than in the normal group (*p* = 0.03).

Among patients in the PAC/PRA ≥ 200 group, 24 received MRA monotherapy. At 3 months, mean blood pressure was significantly reduced compared with baseline: systolic blood pressure decreased from 156 ± 17 to 142 ± 16 mmHg (*p* = 0.0104), and diastolic blood pressure decreased from 94 ± 9 to 85 ± 12 mmHg (*p* = 0.0022).

## Discussion

**Fig. 4:**
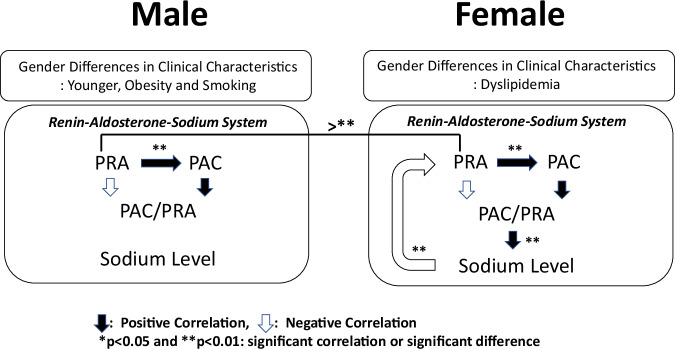
Summary of the study. Closed arrows indicate significant positive correlations (*p < 0.05, **p < 0.001), whereas open arrows indicate significant negative correlations (*p < 0.05, **p < 0.001). PRA plasma renin activity, PAC plasma aldosterone concentration

This study demonstrated gender differences in the renin-aldosterone–sodium system in patients with untreated hypertension, possibly reflecting underlying biological and lifestyle factors. Figure 4 shows a schematic summary of this study, with a focus on gender differences. In this study, we observed gender differences in the clinical characteristics of newly diagnosed hypertensive patients: males were younger and had higher rates of obesity and smoking, whereas females had a higher prevalence of dyslipidemia.

Subsequently, we examined the renin–aldosterone–sodium system. In males, PRA was significantly associated with increased PAC. While higher PAC elevated the PAC/PRA ratio, PRA itself reduced it. No significant correlation was found between the PAC/PRA ratio and sodium levels in males. In females, PRA was also significantly associated with increased PAC. Higher PAC elevated the PAC/PRA ratio, whereas PRA reduced it. Unlike in males, the PAC/PRA ratio in females was significantly associated with higher sodium levels, and serum sodium significantly suppressed PRA. PRA was significantly lower in females than in males; consequently, the PAC/PRA ratio was significantly higher in females. In females, an increase in the PAC/PRA ratio elevated sodium levels, which in turn suppressed PRA, further raising the PAC/PRA ratio. This cycle suggests that the system may play a key role in the early development of hypertension in females. In contrast, in males, the renin–aldosterone–sodium system may play a relatively minor role in the initial rise in blood pressure, whereas lifestyle factors such as obesity and smoking appear to be more important in the onset of hypertension. Although these correlations reached statistical significance, their magnitude was modest. Therefore, the present findings should not be interpreted as demonstrating a strong causal relationship between sodium levels and the PAC/PRA ratio. Rather, they suggest a potential physiological pattern within the renin–aldosterone–sodium system that may differ between men and women. These observations should be considered exploratory and hypothesis-generating, and further studies with direct measurements of sodium intake and more detailed endocrine profiling will be required to clarify the underlying mechanisms.

The concept of low-renin hypertension was originally proposed several decades ago, when it was observed that many hypertensive patients have suppressed renin activity despite elevated blood pressure [10, 13]. These patients frequently exhibit salt-sensitive hypertension and often respond well to diuretics or mineralocorticoid receptor blockade. In this context, our findings may represent a contemporary clinical manifestation of relatively low-renin physiology, particularly in female patients who showed markedly lower PRA levels and higher PAC/PRA ratios in the present study. While these mechanisms are well established, our findings provide contemporary clinical insights into PAC/PRA profiles and sex differences in untreated hypertension, complementing existing knowledge rather than introducing a novel mechanism.

Previous studies have reported that, compared with men, women with hypertension have lower renin activity [14, 15]; however, data on aldosterone levels are lacking. These studies concluded that the cause of this sex difference remains unclear, but they support the hypothesis that the RAAS may contribute to sex differences in blood pressure regulation [13, 14].

Primary aldosteronism is usually sporadic, with its underlying cause being either an aldosterone-producing adenoma or adrenal hyperplasia [16, 17]. In this study, we excluded 28 patients with a high PAC/PRA ratio and elevated PAC who were suspected of having primary aldosteronism, in accordance with the JSH 2019 guideline [6]. These patients accounted for 6.5% of the study population, which is consistent with the previously reported range of 3.2–19.0% [18, 19].

Progesterone increases aldosterone production, whereas estrogen has little effect [20, 21], and testosterone suppresses aldosterone production [22, 23]. Because sex hormone levels decline with age and most patients in this study were over 50 years old, sex hormones are unlikely to have substantially influenced our results. However, future studies should include measurements of sex hormones, such as progesterone and testosterone.

Although aldosterone is primarily produced in the adrenal cortex in response to angiotensin II [18], it can also be synthesized in extra-adrenal tissues independently of renin activity [24–26]. We previously showed that cardiac aldosterone production in hypertensive patients correlates positively with cardiac angiotensin-converting enzyme activity [27], and future studies will examine potential sex differences in extra-adrenal aldosterone production.

Aldosterone plays a key role in the cardiovascular complications of hypertension. Moreover, the detrimental effects of aldosterone and excess salt are synergistic [28–30]. We previously reported that long-term exposure to aldosterone in the presence of elevated sodium levels induces cardiomyocyte hypertrophy via genomic effects mediated by the mineralocorticoid receptor [31]. Therefore, reducing salt intake is recommended for patients with hypertension, particularly those with high PAC/PRA ratio.

According to our results, MRAs significantly reduced systolic blood pressure after three months in patients with a high PAC/PRA ratio. These findings suggest that MRAs may represent a reasonable therapeutic option for hypertensive patients with a high PAC/PRA ratio; however, further large-scale studies are required to confirm these observations. Among patients in the PAC/PRA ≥ 200 group, 24 received MRA monotherapy, and mean blood pressure at 3 months was significantly reduced compared with baseline. The marked blood pressure–lowering effect of MRA monotherapy suggests that aldosterone-dependent mechanisms may play a major role in the pathogenesis of hypertension in these patients, potentially reflecting subclinical hyperaldosteronism or salt-sensitive hypertension. These observations are consistent with earlier reports indicating that patients with suppressed renin activity may respond preferentially to therapies targeting sodium retention or mineralocorticoid signaling. Our findings extend these observations by demonstrating that stratification using the PAC/PRA ratio in untreated hypertensive patients may help identify individuals who benefit from early MRA therapy.

In our treatment protocol, initiation of MRA was recommended for patients in the high PAC/PRA group unless contraindicated. Although MRA was initiated or considered in all eligible patients at baseline, ~20% were not receiving MRA at 3 months. Discontinuation or non-use were attributable to clinical factors, including the development of hyperkalemia, a decline in renal function, patient intolerance (e.g., gynecomastia, dizziness, or erectile dysfunction), physician judgment based on comorbidities or blood pressure response, and patient preference. The 2025 JSH guidelines recommend a target blood pressure of <130/80 mmHg [7]. Accordingly, many patients in this study will require further blood pressure reduction through lifestyle modification and/or pharmacological therapy.

Importantly, the cut-off value used in the present study should not be interpreted as a biologically definitive threshold. Plasma aldosterone concentration, plasma renin activity, and their ratio are inherently continuous variables. The threshold applied in our analysis was introduced as an exploratory and pragmatic approach to stratify patients according to the treatment protocol. Therefore, this value should be interpreted cautiously and viewed primarily as a clinical stratification tool rather than a strict biological boundary.

This study had several limitations. It included a small number of patients and lacked data on sodium intake and excretion, angiotensin, angiotensin-converting enzyme, the autonomic nervous system, and atherosclerotic factors. Larger-scale studies are needed to explore these fundamental aspects. Recent reports have suggested that chemiluminescence enzyme immunoassay (CLEIA) methods are reliable alternatives to radioimmunoassay (RIA) [32]. However, another study also demonstrated a significant correlation between PAC values obtained by RIA and those obtained by CLEIA [33]. Further research using CLEIA is warranted.

### Asian perspectives

In Asia, particularly in countries such as Japan, high dietary sodium intake and a higher prevalence of salt sensitivity are well-recognized characteristics of hypertensive populations, often accompanied by suppressed plasma renin activity and a predominance of low-renin hypertension. However, our study extends this framework by highlighting the clinical relevance of not only low-renin profiles but also relatively elevated plasma aldosterone concentrations. In this context, our findings suggest that stratification based on both plasma aldosterone concentration and plasma renin activity may provide a practical approach for tailoring antihypertensive therapy in Asian patients. While low-renin individuals may benefit more from natriuretic or mineralocorticoid receptor–targeted therapies, those with relatively higher aldosterone levels may represent a subgroup in whom aldosterone-mediated mechanisms play a greater role, even in the absence of overt primary aldosteronism.

## Conclusions

We identified gender differences in the renin–aldosterone–sodium system in untreated hypertensive patients. MRAs were effective in lowering blood pressure in hypertensive patients with a high PAC/PRA ratio who did not have primary aldosteronism, as well as in those with low PRA levels.
